# Piloting a Home Visual Support Intervention with Families of Autistic Children and Children with Related Needs Aged 0–12

**DOI:** 10.3390/ijerph20054401

**Published:** 2023-03-01

**Authors:** Marion Rutherford, Julie Baxter, Lorna Johnston, Vaibhav Tyagi, Donald Maciver

**Affiliations:** 1 School of Health Sciences, Queen Margaret University, Edinburgh EH21 6UU, UK; 2 NHS Lothian Speech and Language Therapy Department, Edinburgh EH16 4TJ, UK; 3 City of Edinburgh Council, Additional Support for Learning Service, Edinburgh EH8 8BG, UK

**Keywords:** assessment, visual supports, autism, parents, home

## Abstract

Visual supports are an important intervention for autistic individuals and others with neurodevelopmental differences. However, families often report limited access to visual supports and lack of information and confidence in their use at home. This pilot study aimed to evaluate the feasibility and effectiveness of a home-based visual supports intervention. Methods: 29 families with children (n = 20 males; mean age 6.59 years [Range 3.64–12.21 years SD 2.57]) receiving support for autism or related needs participated in the study. Parents engaged in an individualised assessment and intervention process through home visits, completing pre- and post-measures. Qualitative methods were used to explore the parents’ experiences of the intervention. Results: The intervention led to a statistically significant improvement in parent-reported quality of life (t28 = 3.09, *p* = 0.005) and parent-reported perception of autism-specific difficulties (t28 = 2.99, *p* = 0.006). Parents also reported improved access to resources and relevant information and increased confidence in using visual supports at home. The home visit model was strongly supported by the parents. Conclusion: The results provide initial evidence of the acceptability, practicality, and utility of the home-based visual supports intervention. These findings suggest that outreach into the family home may be a beneficial mechanism for delivering interventions related to visual supports. This study highlights the potential of home-based interventions to improve access to resources and information for families and the importance of visual supports in the home setting.

## 1. Introduction

There is no single intervention recommended for all autistic individuals; however, some interventions are effective across several situations [1,2]. One such intervention is the use of ‘visual supports’. The term visual supports describes a range of tangible, relatively low-cost resources, relevant to a range of developmental levels, such as objects, photos, and picture symbols, which are used to support receptive or expressive communication and to help reduce anxiety, increase predictability, and support understanding of routines and social expectations [3]. Visual supports are commonly used by autistic individuals and by individuals with other communication or neurodevelopmental differences [1,2]. They are typically integrated into a range of interventions at home [4] or in schools [5]. Environmental adaptations [6,7,8], and specifically visual supports [1,2,9] are commonly highlighted in reviews and often recommended in good practice guidelines.

The lack of formally published visual support resources for use by parents motivated us to complete the current study, with a gap identified in the evidence available about the use of visual supports by families at home [3]. There is persuasive evidence that both practitioners and parents would like more training in the use of visual supports and that a structured approach to training and coaching can improve knowledge, skills, and outcomes [10]. Where this study was completed (Central Scotland, United Kingdom), visual support training is available to all nursery and primary schools [11], and visual supports are commonly used in these settings. Although parent interventions, parent training, and information related to autism are also available, families reported a gap in the provision of home-based visual supports [3]. Parents also reported learning about visual supports in parent groups but that transferring this knowledge to daily life is challenging [3].

The purpose of this study was to pilot a Home Visual Support intervention with families of autistic children and children with related neurodevelopmental differences aged 0–12. This study took place in a city in Scotland with a population of 500,000, where around 400 children, aged 0–18 years, are referred for autism diagnostic assessment annually [12]. In Scotland, most autistic children attend their local mainstream school or nursery. Integrated support is offered through multi-agency teams of health, education, social care, and third sector partners. Support is intended to be based on need not diagnosis, in line with local legislation. Neither a pre-specified number of hours of treatment nor behaviourist support are generally offered in Scotland, as this is not in keeping with the needs and wishes of the autistic community. A range of universal, targeted, and specialist supports are provided, according to need, developmental stage, and taking account of adaptations to naturally occurring environments. This includes collaborative approaches between health and education practitioners, parent mediated interventions, parent information sessions, and training, modelling, and coaching with staff in education settings.

## 2. Methods

### 2.1. Research Question

Our main research question was focussed on understanding to what extent a model of three to five home visits was effective in improving parent quality of life and in improving parent confidence and knowledge in use of visual supports. We hypothesised three to five home visits would be sufficient to deliver the intervention, and that parents would value this method of intervention delivery. We hypothesised that there would be an increase in parent quality of life associated with the intervention, and an improvement in parent confidence and competence in using visual supports.

### 2.2. Participants and Procedures

Locally, children are assessed for autism through following the local National Health Service (NHS) diagnostic pathway. This involves assessment by a multi-disciplinary NHS team, following SIGN (2016) clinical guidelines and standards [1]. However, autistic people and their families often express an aspiration that support should be needs-led, not diagnosis-led. We therefore made the decision to extend study inclusion to those undergoing assessment for autism as well as those diagnosed. The study was open to parents/carers of children with diagnosed autism or related support needs, whose children were aged 0–12 years old, residents in the city, and referred from an additional support for learning (special education support) central service for the city because they were autistic or autism was suspected. Parents were recruited through direct approach from the project team. Parents were called by phone and then sent written information and asked to sign a consent form. The project team made contact with the family to begin involvement on receipt of consent forms. Families were volunteers and were free to withdraw at any time for any reason. This work was carried out in accordance with the relevant ethical standards of institutional and national practice in Scotland, and was in-line with the Declaration of Helsinki. Approval for this project was provided by the research access service for the City of Edinburgh local authority (local government) research access service.

### 2.3. Visual Supports Intervention Model for the Current Study

There was no readily available comparable resource pack or intervention focussing on home visual supports. We opted to develop our own and then to undertake this study. Based on an iterative process of literature review and consultation with parents and a multi-disciplinary group of educationand health professionals, we developed the ‘Home Visual Support Intervention’. This was designed to be a brief intervention that we hoped could be readily integrated into routine practice to address the difficulties families have in accessing and using visual supports resources. Resources developed included a staff training pack and guidance; visual support symbol resources in electronic format with written guides to inform how each was used; and assessment tools—each of which were available to download online. The intervention, which was aimed at families of autistic individuals, was designed to be based on individual needs rather than diagnosis, and was intended to be integrated into existing support services for people with various communication support requirements. The underpinning model applied the following principles:

1. Families are equal partners;
2. Support should be provided to enable families to make visual supports resources independently;
3. Assessment and planning processes are multi-disciplinary, including health and education staff, dynamic, parent focussed, and developmentally and functionally relevant;
4. Support is ‘time friendly’ with long-term access to visual supports for professionals and parents;
5. Family friendly information and signposting is provided;
6. Flexibility of access to professional support is provided to include intensive start-up options, drop-in sessions, peer support, home support, parent training, coaching, and modelling;
7. Professional training and awareness raising around visual supports are included;
8. There is consistency across settings with ‘symbol sets’ and templates of commonly used resources.

Resources focussed on visual supports relevant to three developmental levels, as specified by Prizant et al. (2003) [13]. These were visual supports targeted to (1) ‘Social Partners’ (children with less than 10 words), (2) ‘Language Partners’ (children with short phrase speech), and (3) ‘Conversation partners’ (children who can engage in extended conversational interactions) [13]. The content included a practitioner’s resource pack, a parent self-assessment tool, and a visual symbol selection tool, in order to support decisions about which were relevant to current needs at home and which particular symbols to use. Detailed ‘how-to guides’, FAQs, and sets of instructions were written for the visual supports and how to use each. The specific visual supports used with each family were selected based on the individual child stage (as defined in terms of the child’s developmental communication level [13]) and family context (see Table 1). The intervention was designed to be delivered by staff with pre-existing knowledge of autism and visual supports, and completed over three to five visits to the family home.

### 2.4. Staff Providing the Intervention and Intervention Delivery

Staff providing the intervention had relevant prior knowledge and training. Staff with ‘enhanced’ or ‘expert’-level skills [14] in autism and visual supports were used. In total, the intervention was delivered by six professionals, including education practitioners, specialist project workers, and speech and language therapists. All staff received the staff training pack, guidance, and resources prior to implementing the intervention. Implementing staff attended a 2 hour training. To deliver the intervention, families were visited three to five times at home, with activities as follows:

- Prior to Visit 1: Parent completed the pre-questionnaire and assessments. These were subsequently collected on Visit 1. Support to complete these was offered;
- Visit 1: Parent assessment was completed to identify priorities relevant to child’s stage and family targets. Between visits, staff collected visual supports resource pack for the family and prepared ‘how to’ guides. Staff could make individualised resources if required. A local ‘Boardmaker in Libraries’ programme [15] was also available to families to attend ‘drop-ins’ or to make their own resources. Families were made aware of this, although use and experience of this external maintenance mechanism was not evaluated in this study;
- Visit 2: The professional brought agreed visual supports and provided guidance on using them (written guides, modelling, coaching, and verbal guidance). Families had the opportunity to ask questions, and in partnership, adjustments were made to the plan. If necessary, further assessment of need could be completed, and more visual supports identified;
- Visits 3–5: Parents used visual supports and provided feedback. Staff offered further consultation and brought additional visual supports if needed. On completion, staff provided a summary of visual supports implemented and guidance shared, which the family can share with school or use as a reminder;
- After the final visit: Parents were given the post questionnaires/assessments at the end of the final visit. These were collected by a team member at a suitable time within a week of the last visit.

### 2.5. Measures

#### 2.5.1. Outcomes Model

Parent quality of life and parent confidence and knowledge in use of visual supports were the main outcomes. In research aimed at understanding which interventions are effective for autistic people, there is a shift of emphasis towards the importance of wellbeing outcomes, and towards those that consider the family context [16,17,18]. Quality of life (QoL) is a developing focus [19]. There is strong evidence of lower QoL in autistic people and their families [20,21,22]. Measures specific to autism are in development [17,19], and autism intervention studies are beginning to use QoL measurement tools.

#### 2.5.2. Parent Assessment Tool

A Parent assessment tool was designed for this project and used to identify targets around use of visual supports and the families’ perceived needs. This tool includes questions for focussed discussion between parents and professionals to establish the child’s communication stage, parent’s previous experience of using visual supports with their child, and to clarify whether there are current aspects of home life that would benefit from visual supports. It takes 45–60 min to administer and generates 1–3 targets per child.

#### 2.5.3. The Quality of Life in Autism Questionnaire (QoLA)

The QoLA [20] was used pre- and post-intervention. The QoLA is two-part questionnaire. Part A contains 28 items designed to measure overall perception of QoL, and Part B contains 20 items designed to measure how problematic a child’s autistic symptomatology is. In Part A, parents rate their agreement on items on a scale of 1 (‘Not at all’) to 5 (‘Very much’). In Part B, parents rate problems caused by difficulties experienced by autistic children on a scale of 1 (‘Not much of a problem’) to 5 (‘Very much of a problem’). Internal consistency coefficients for the QoLA are α = 0.94 for Part A and α = 0.92 for Part B for parents of autistic children [20].

#### 2.5.4. Parent Questionnaire

A parent questionnaire was used pre- and post-intervention. This was a bespoke set of questions devised to consider parents’ self-assessed level of knowledge, skill, and understanding of how to use visual supports with their child. The parent questionnaire was undertaken as part of the intervention and took between 30 and 40 min.

### 2.6. Data Analysis

Paired *t*-tests were performed on pre- and post-intervention scores for the QoLA to assess the effect of the intervention. Significance was set at *p* < 0.05. Missing data were low, and any missing values were imputed through the multiple imputation procedure (Markov chain Monte Carlo). Prior to conducting paired *t*-tests, we conducted Shapiro–Wilk tests to test for assumption of normality—the differences of paired data showed normal distribution for part A (W = 0.97, *p*-value = 0.69) as well as part B (W = 0.93, *p*-value = 0.07) of the QoLA. Demographics and parent questionnaire data were summarised and presented descriptively.

### 2.7. Qualitative Data Collection

Parents were invited to attend a focus group or individual interview. Of the families who completed their involvement at the 6-month point of the project, four agreed to attended a focus group and one attended an individual interview. Participants were asked the same questions across groups and interviews. Questions aimed to elicit discussion on (1) how visual supports were used in the home setting (e.g., how did you first find out about visual supports and how they can be used? Which visual supports do you use most at home—which ones work well? What did not work well when you used visual supports at home?) and (2) how they were supported by professionals to do this (e.g., think about the support that you were offered, and what could be done differently or improved on? What would an ‘ideal package’ of help and support to families look like? What information, resources and/or training opportunities would you like to have access to?). Focus groups were 90 min maximum and interviews were 60 min maximum. Two impartial members of staff, who were not part of the intervention or research team, completed the focus groups and interview.

### 2.8. Qualitative Data Analysis

Data were transcribed verbatim and thematic analysis undertaken [23]. To ensure rigour and trustworthiness, coding was completed and cross checked by two researchers. The findings were further reviewed and themes debated through discussion with team members and colleagues. Themes were identified with an audit trail maintained thorough direct parent quotes.

## 3. Results

Data were collected for 29 children and their families. Participant demographics are outlined in Table 2. The average time between pre- and post-assessment was 52 days (min = 21 days, max = 96 days). Each family received a minimum of three and a maximum of five visits.

### 3.1. Pre-Post Intervention Changes

**Figure 1:**
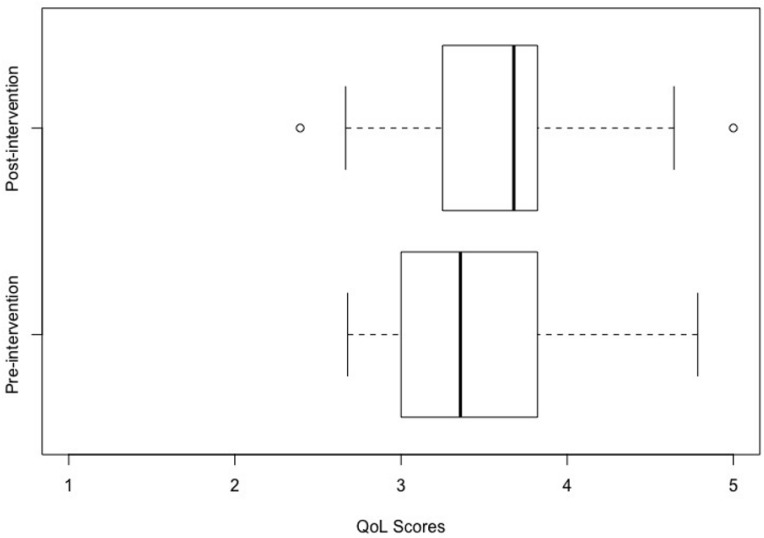
Effect of intervention on perceived quality of life scores. Intervention (binary variable i.e., pre- and post-intervention) is plotted on the y-axis, while QoLA-A scores (range: 1 to 5) are plotted on the x-axis. Note: Higher scores on the x-axis reflect better perceived quality of life.

**Figure 2:**
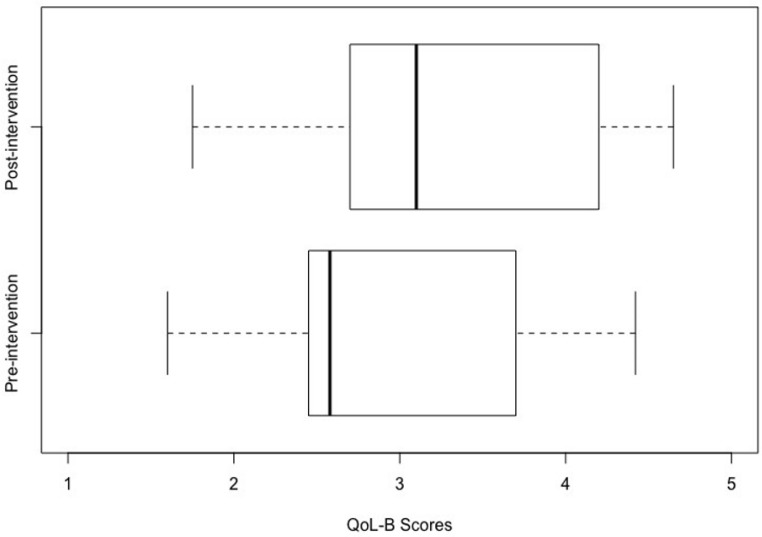
“Effect of Intervention on perceived autism specific difficulties scores”. Intervention (binary variable i.e., pre- and post-intervention) is plotted on the y-axis while QoLA-B scores (range: 1 to 5) are plotted on the x-axis. Note: Higher scores on x-axis reflect fewer perceived autism specific difficulties.

Following intervention, there was a statistically significant improvement in parent-reported QoL (t(28) = 3.09, *p* = 0.005) and a statistically significant reduction in parent-reported perception of autism specific difficulties (t(28) = 2.99, *p* = 0.006) (Table 3) with moderate effect sizes for both (Cohen’s d > 0.5). The box plots in Figure 1 and Figure 2 demonstrate the distribution of QoLA-A and QoLA-B scores (Figure 1 and Figure 2) on a scale of 1 to 5. Higher scores on QoLA-A show greater perceived QoL. Higher scores on QoLA-B show fewer perceived autism-specific difficulties. The whiskers on each side represent the minimum and maximum values in the datasets, while the lower and upper quartiles are represented by the boxes. Despite a small sample size, the dataset was homogeneous in terms of parent-reported QoLA-A and QoLA-B scores. This is evident in box plots presented in Figure 1 and Figure 2 where the boxes are narrow (between average scores of 3 and 4 for QoLA–A and between average scores of 2.5 and 3.75 for QoLA–B on scales of 1 to 5).

### 3.2. Parent Questionnaire

The results of the questionnaire, compared pre- and post-intervention, revealed that parents reported a significant increase in their knowledge about visual supports, access to such supports, and their confidence in using them. Prior to the study, 43% of parents previously used visual supports at home and 57% previously received information and support in relation to visual supports. After the project 100% used visual supports and received information, support, and visual support resources. Parents were asked to rate the support they received, their confidence now and for future use of visual supports, the amount and usefulness of information, their knowledge, and their ease of access to visual supports. On all of these measures, there were improvements for all families, as shown in Supplemental File S1.

### 3.3. Qualitative Findings

Five themes were identified.

**Theme 1: The home visit mechanism was highly valued by parents**

Parents had strong feelings about the intervention and particularly the beneficial nature of visits at home and how this was more useful than previous ways of receiving information. Parents were highly motivated to make adaptations to their daily routines to support their children. Parents reported that they experienced many stressors and difficulties in their lives, but despite this, they were still eager to make changes to help their children. The families appreciated the home visits that they received as part of the intervention, which was a marked difference from previous ways of receiving information.

*“I have never been offered visuals by the school for use at home. Only since the [staff] came to visit did I really get it.”*

*“What I liked was the way they came back, so that we were able to tailor it to our current circumstances and then follow it up.”*

*“We did parent groups… the experience of someone coming into your own home was quite different”*

*“It was different from other experiences of Visual Supports.”*

Parents reported that the home visits allowed staff to better understand the family’s context and needs and to provide tailored support. The staff were seen as positive, supportive, and empathetic in their interactions with the families. They asked good questions, listened to the parents’ needs, and provided feedback and encouragement. As a result, the parents felt empowered and more confident in their ability to support their children.

*“It was better for them (staff) to actually see things (at home), it gave it all context. There are real benefits to them coming to the home.”*

*“I had bought Visual Supports online (before) but wasn’t really using it. The [staff] helped me use it effectively.”*

*“They asked really good questions, they could really help… made us realise that it doesn’t need to be like this.”*

*“The [staff] workers never said you’re doing it wrong. It was so comforting to have strangers tell you that you are doing great.”*

The families appreciated the individualised nature of the support and the chance to participate in planning and making decisions about the visual supports. This allowed them to feel more involved and invested in the process, which helped to increase their confidence and motivation to support their children.

*“We came up with the ideas together.”*

*“The [staff] team really listened and I found it encouraging. It pushed me more to help my child.”*

*“They answered all my questions.”*

*“Crucially, I got to choose.”*

Overall, the families reported that the home visits were a valuable and effective way to receive support and information about visual aids. The tailored, individualised approach was seen as much more beneficial than group settings, and the families felt that they had more control over the process. The visits provided a positive and supportive environment that allowed the families to feel listened to and encouraged.

**Theme 2: Individualisation, tailoring, and developmentally appropriate support is valued by parents**

The benefits of having specific and tailored support was a dominant theme among all the families. Participants were pleased with the level of support that was provided to them and appreciated the personalised nature of the support. They noted that an individualised approach helped them, as a family (including siblings), to understand and effectively utilize visual supports. Moreover, the children themselves were involved in the decision-making process to personalize the supports, which led to an increased level of engagement and ownership. Parents described the amount of support as optimal, and noted that the support was beneficial for them, for example, in reducing their anxiety.

*“The amount of support was just right.”*

*“I was very anxious but this experience was so personal and focussed on my own needs, that was nice.”*

The participants emphasised the value of having tailored support that was focused on their specific needs, as opposed to a generic approach that was taught in a group setting. They felt that this type of support was more valuable and beneficial to them, as it was centered on their individual concerns and issues. Additionally, the participants appreciated the opportunity to have one-on-one time for specific advice, which was deemed to be more helpful than receiving advice in a group setting.

*“Teaching us about it individually is much better than teaching in a group. It is individualised and personalised.”*

*“I liked how personal it was.”*

*“Groups are not about your child but [the project] was about our child.”*

*“Time for specific advice for me rather than in a group is so much more valuable.”*

An individually tailored approach supported parents and siblings to understand and use visual supports effectively. The families had children at different stages of communication and were positive about the supports that were tailored to their child’s specific stage of development. Parents appreciated that the support was set up for their child as an individual and that the progression was explained in a way that was appropriate for their child.

*“He didn’t have symbolic understanding, so it was tailored to his needs… It was set up for him as an individual and the progression to symbols was explained.”*

This individualised approach helped the families to make progress and achieve their goals, and was widely regarded as a critical aspect of the success of the intervention. Children also took part in the decision making to personalise supports, which families viewed as beneficial.

*“[My child made the] choice of what to focus on and symbol selection.”*

*“My daughter has made her own visuals.”*

**Theme 3: Timing is important**

The theme of timing was identified as a critical factor in the effective use of visual supports by parents. Three key aspects of timing were identified: early or timely access to visual supports, appropriate duration of support, and maintenance of the support over time. Participants emphasised the importance of clear expectations at the start, which helped them feel satisfied with the level of input they received. They also acknowledged the benefits of early access to expertise and the role that follow-ups play in providing support during key times, such as transitions.

*“The sooner you have access to expertise and from as young an age as possible, the better.”*

*“If this had been introduced to me 2 years ago, I would have needed much more support.”*

*“Eventually we will get better but we need lots of input.”*

*“Follow ups are crucial at key times (e.g., phone calls at times of transition).”*

Establishing clear expectations from the outset provided a sense of comfort for parents, as they felt informed about the level of involvement required. The participants observed that although support was essential, they did not expect to require it indefinitely. 

**Theme 4: Parents report multifaceted benefits to visual supports at home**

A range of benefits were reported with regards to understanding the full range of what visual supports were capable of doing. Parents particularly reported increased knowledge and understanding of the capabilities of visual supports, beyond their initial perception of them as ‘just timetables’. They found that visual supports provided more than just a visual representation of routines, and the intervention gave them a deeper understanding of the possibilities for visual aids.

*“I didn’t realise the scope of [visual supports] before. A lot more info would be useful to parents.”*

*“Visuals helped immediately–it was like, really? That’s all it takes?”*

*“The [visual supports] has helped, it’s so much more than just timetables.”*

*“The Nursery was always supportive in using visuals but it was really timetable based. I found using these difficult as home is different than school. However now I understand that it’s about so much more than timetables. I have a much broader understanding.”*

In addition, families reported increased independence in their children, with some children setting routines for themselves and taking more control over their schedules. This was seen as a positive outcome, as it not only helped with consistency, but also encouraged independence.

*“One morning my child got up and set the timetable himself.”*

*“We wanted our son to be more independent and the [visual supports] has helped.”*

*“It can help behaviour but also encourages independence.”*

*“I do believe it has helped keep his behaviour more consistent at home.”*

Furthermore, the use of visual supports provided families with access to resources and information, making them feel informed and equipped to tackle new challenges. Sharing information with other families and learning from each other was also seen as a benefit, with parents feeling inspired by the experiences of others.

*“I want to share my story to inspire others.”*

*“It felt good to know that other families were experiencing the same thing.”*

*“I’m thinking creatively now and I have ideas for tackling other issues.”*

Finally, visual supports were seen as beneficial in terms of helping children develop skills, particularly in communication, through consistency in words and routines. Parents saw the use of visual supports as a tool that provided numerous benefits, helping them to tackle difficult challenges and improve the skills and independence of their children.

*“It has helped me tackle hard things.”*

*“We became more consistent with words we used and the order we did things, his speech has really improved.”*

**Theme 5: The challenges of visual supports**

While the majority of parents expressed positive views towards the use of visual supports, they were also aware of the limitations and challenges associated with this approach. Parents recognised that visual supports are not a one-size-fits-all solution, but rather a tool that needs to be tailored to each individual child and situation. Parents noted that visual supports were not always practical, especially for those with busy schedules, and that not all approaches were effective for all children. However, they emphasised that the challenges were not significant enough to outweigh the benefits of using visual supports, and that they gained valuable insights into new ways and places to use visual supports effectively.

*“It’s not been magical but it helps.”*

*“Visual support is great but is does have its limitations.”*

*“If you are busy it’s not always practical.”*

The overall conclusion from the parents was that visual supports were a useful tool, but one that had limitations, and that continued experimentation and adaptation were necessary to find the most effective approach.

*“Not everything worked but I have ideas of other ways and places to use visual supports.”*

### 3.4. Summary of the Qualitative Analysis

Regarding the home visit mechanism, parents reported feeling listened to and positively engaged with during visits from support staff. They valued the opportunity to receive information and support in their own homes, as it provided context and a personalised experience compared to previous methods of receiving information. They appreciated the opportunity to participate in the planning process and receive positive feedback from the staff, leading to feelings of empowerment and confidence. The individualisation and tailoring of support was also highly valued. Parents appreciated the personal and focused approach that was tailored to their own needs and those of their children. They reported positive outcomes in terms of understanding and effectively using visual supports, as well as their children being involved in the decision-making process to personalize the supports. Parents were also positive about supports being tailored to their child’s stage of communication.

Timing was another key theme that emerged, with parents reporting the importance of early and timely access to visual supports, the appropriate duration of support, and the need for follow-up maintenance. They emphasised that the sooner they had access to support, the better, and that follow-ups at key times, such as during transitions, were crucial. Parents also reported a range of wide benefits from using visual supports at home. They reported increased knowledge and understanding of the capabilities of visual supports, and appreciated the support being broader than just timetable-based.

## 4. Discussion

In a pilot study involving families of 29 children, a novel visual supports intervention was found to be feasible and effective. Experienced staff were able to deliver the intervention in three to five home visits, using resources that were relevant and accessible to parents. The approach required individualisation for each family through assessment. Pre-prepared packs and guidance were time saving for staff and the assessment structure facilitated comprehensive discussions. The home visit mechanism was reported to be very beneficial by parents.

The families completed the QoLA, a measure of quality of life, which revealed a statistically significant improvement after the intervention. Families also reported an increase in their confidence and knowledge to use visual symbol supports, as well as improved access to visual support resources. However, they did not view visual supports as a panacea, but rather as a useful tool to increase predictability, understanding, and desirability of home and community activities.

Families gave feedback in relation to types of visual supports that are useful at home and highlighted that visual timetables are more difficult to use at home, which is less structured than school. A range of visual supports were found to be helpful in planning routine and non-routine events, such as such as shopping trips, holidays, transitions or new activities. Notably, the individual visual supports introduced in the study were not novel. Rather, the addition provided by this approach was a framework that structured the process of assessment and decision-making, combining professional knowledge of the approaches and developmental expectations with parent knowledge and experience.

Overall, the intervention created access to the supports most useful at a point in time, and knowledge about how to use them, with support to start and adapt their use. To our knowledge, this is the first intervention study to use the QoLA as an outcome measure, showing positive results that are reflected in the qualitative data. Our findings suggest that this intervention has the potential to improve the quality of life for families, and warrants further investigation in larger studies.

Our study adds to an emerging body of home and community visual supports research [3]. In previous studies, visual supports were used successfully; however, the number and quality of studies limits the possibility of drawing strong conclusions. While a body of research is present on different kinds of visual supports for autistic children and families [9], complexities of provision and application alongside indicated that realistic, straightforward strategies were required. The dynamic nature of successful implementation of visual supports makes this a challenging area to research. There is a requirement for consensus in terminology, assessment/planning protocols, and tools. It was previously shown that parents are more likely to invest time and effort and to experience success with home visual supports if they have access to clear information and evidence about how to use them [24]. Accessibility is a key feature. It was identified that parents value access to training, resources, and support [25]. Other research concluded that developing a “bank” of commonly used visual supports is beneficial and time efficient [24] e.g., ‘Boardmaker in libraries’ [15].

Studies consistently highlight the need for visual supports to be integrated alongside parent and child goals [26]. Good partnership working and collaboration with parents is of key importance. Highly skilled teams with several different professionals are recommended, as professional–family collaboration is a known area of difficulty [27]. Additionally recognised in other research is individualisation and appropriate supports for the developmental level; this is of key importance to parents and children alike [28].

Finally, working in a naturalistic context (the home) is cost-effective [29]. Families may be ‘time poor’; so, the time requirements for parents also needs to be considered. There is a wider body of research showing parents benefit from systematic instruction at home, and this evidence supports the benefits of our home programme [30]. The need for new assessment and planning tools specifically for visual supports was also highlighted in the literature [3].

From a professional’s perspective, visual supports are unlikely to be used as the only support or strategy in place, and it is important to ensure they are meaningful and desirable to the individual and to integrate their use well with other approaches in place [26]. Working within a neurodiversity paradigm is increasingly important in relation to all support and intervention for autistic individuals [31]. The same supports can be used in different ways, but through this paradigm, the focus is on valuing diversity and using visual supports to facilitate meaningful engagement and participation, seeking, in particular, acceptability to the autistic community [32] and supporting shared understanding. 

Time taken to learn to use a visual support varies as a result of many factors. The challenge of ‘teaching’ individuals to use visual supports at home is reduced when these are at the right developmental stage and are meaningful; however, just putting the visual supports near the child might not lead to them knowing what to do. Some learners may need adults to use coaching methods [33] to model, cue, prompt, and provide repeated meaningful opportunities for them to see the benefit of them and use them [34]. Where things do not go smoothly, parents are more likely to give up without access to professional support and troubleshooting to understand how they might adapt their approach [3]. The more the supports are used in the environments in which they are relevant, the more likely they will be used, and if they are used across settings, this will lead to greater consistency. For older children and young people, self-management of visual supports is encouraged [35].

### 4.1. Limitations

In terms of limitations, this is the first study of this intervention and the sample size is relatively small. We cannot be certain which elements of the intervention were effective, and there was no control group. Replication studies would improve confidence in the effectiveness of the intervention. There are, as of yet, no long-term outcome measures to evaluate the effects of the intervention over time. The inclusion of children undergoing assessment, as well as after diagnosis, could be seen as a limitation and may prompt further debate; however, professionals working across health and education are now encouraged to offer early intervention and to support need, not diagnosis. This is certainly a message strongly received from families, who indicated that they do not want these environmental supports to be kept from them until diagnosis is made; therefore, the findings could be seen as being relevant to and respectful of parents of children who do not fall neatly into categories.

### 4.2. Implications for Practice

Based on the parent consultation and the review of the outcomes of the study, recommendations are made below for the application of visual supports intervention with home visiting.

1. Home visits can be used as an effective way of providing brief interventions focussed on visual supports;
2. Parents can take positives, even when things do not work—so several symbols and methods should be tried;
3. Professionals should listen to families and their needs and set appropriate expectations about the level of input and likely benefits of visual supports;
4. Professionals should offer visual support as early as possible, and help parents find it, understand it, and use it;
5. Parents report positively when they have access to a range of resources relevant to a range of developmental levels;
6. Parents should be involved in the assessment process and selecting the symbols used from an early stage;
7. It is helpful when staff model how to use visual supports in context (i.e., at home);
8. Individualisation and tailoring are viewed positively by parents, particularly around the child’s individual developmental level;
9. Follow up visits are needed with parents with the chance to review and adapt, and maintenance options for symbols and other technology are helpful, though these should not be too complex;
10. Enhanced knowledge and skills to assess needs and implement visual supports are a core practice for specialist educational professionals and some allied health professionals (for example occupational therapists and speech and language therapists). Such professionals would be well placed to apply home visual support interventions.

### 4.3. Implications for Research

We make a number of recommendations for further study. First, a replication study with a larger sample would be beneficial. Second, the resources can be made available online, with feedback gathered on the utility of this delivery mechanism. Third, we aim to develop and evaluate professional learning for staff delivering visual support interventions for families in the home setting. Lastly, further consideration is required of neurodiversity affirming [36] use of visual supports and their acceptability to autistic people. A ‘deficit’ lens using pejorative or problem-based language is now seen as shaming and pathologising, meaning careful review and co-production with neurodivergent people and families is required to make assessments and supports that do not generate negativity and distress.

## 5. Conclusions

Visual supports are an efficient, low-cost intervention. This study describes development and piloting of a novel visual supports interventions for families of autistic children and those with related neurodevelopmental differences. The study identified that parents can effectively be included in a visual supports intervention at home. A structured approach to implementing visual supports is effective in increasing parent-reported QoL, and need not be time intensive when pre-prepared resources are available.

## Figures and Tables

**Table 1:** Visual support resource bank available to staff delivering the intervention.

Resources for Child to Use by Stage	* Social Partners	* Language Partners	* Conversation Partners
Object of reference (e.g., spoon to represent meals)	✓		
This is what we are doing ‘Now’ object of reference	✓		
Now/next board and objects	✓		
Object timetable for part or all of the day	✓		
Song signifiers (objects of reference)	✓		
Choice board	✓	✓	✓
Personal photo book	✓	✓	✓
Technology-based (e.g., computer or touch device)	✓	✓	✓
Now/next board and symbols		✓	
Picture exchange communication system		✓	
Sequence chart for daily routines (e.g., toileting)		✓	✓
Countdown cards (e.g., 3 sleeps until …)		✓	✓
‘Help’ card		✓	✓
‘Wait’ card		✓	✓
Song symbol book		✓	✓
Visual symbol shopping list		✓	✓
Visual/symbol timetable for part or all of day		✓	✓
Visual timetable or calendar for the week/month			✓
‘OK/Not OK’ cards			✓
Emotions (e.g., emotions regulation visual)			✓
Social Stories			✓
**Resources for parents/environment at any stage**			
Home/school visual diary			
Communication passport			
Environmental visual labelling to show where things are and what goes where (e.g., photo above coat)			
Sand timer to prepare for transitions/learning to wait			

* As defined in Prizant et al., 2003 [13].

**Table 2:** Participant demographics (n = 29).

**Child Age (Years)**	Mean (SD) 6.59 (2.57)	Range 3.64–12.21
**Parent Age (Years)**	Mean (SD) 36.79 (6.23)	Range 24–48
**Child Gender**	**N**	**%**
Male	20	68.97
Female	9	31.03
**Number of Siblings**		
1	19	65.52
More than 1	10	34.48
**Diagnosis of Autism**		
Yes	21	72.41
No	8	27.59
**Use Visual Supports at home**		
Yes	8	27.59
Unsure	12	41.38
No	8	27.59
Missing	1	3.45
Current IEP *		
Yes	4	13.79
Unsure	6	20.69
No	18	62.07
Missing	1	3.45

* Individualised educational programme, denoting extra support in the school context. Note: all participating parents were mothers.

**Table 3:** Paired *t*-tests on QoLA pre- and post-intervention (n = 29).

Measure	t	df	*p*	95% CI	Mdiff	Effect Size (Cohen’s d)
A. Quality of life	3.09	28	0.005	[0.067, 0.331]	0.199	0.573
B. Autism specific difficulties	2.99	28	0.006	[0.127, 0.680]	0.404	0.556

## Data Availability

Please contact the corresponding author for study data.

## Supplementary Materials

The following supporting information can be downloaded at: https://www.mdpi.com/article/10.3390/ijerph20054401/s1, Additional file S1: Parent questionnaire pre-post data summary.
