# Supplementary material for: Piloting a Home Visual Support Intervention with Families of Autistic Children and Children with Related Needs Aged 0–12
Source: Int J Environ Res Public Health. 2023 Mar 1;20(5):4401. doi: 10.3390/ijerph20054401 (PMC10001844; doi:10.3390/ijerph20054401)
Supplement: Supplementary file 1 [file ijerph-20-04401-s001.zip › ijerph-2084559-supplementary.pdf]

Supplemental files

Additional file S1: Parent questionnaire pre-post data summary

Additional Figure: Parent pre-post questionnaire results, mean (average) scores (n=29)

Item score range 1-5, 1-4 and 1-3 (see below for key)

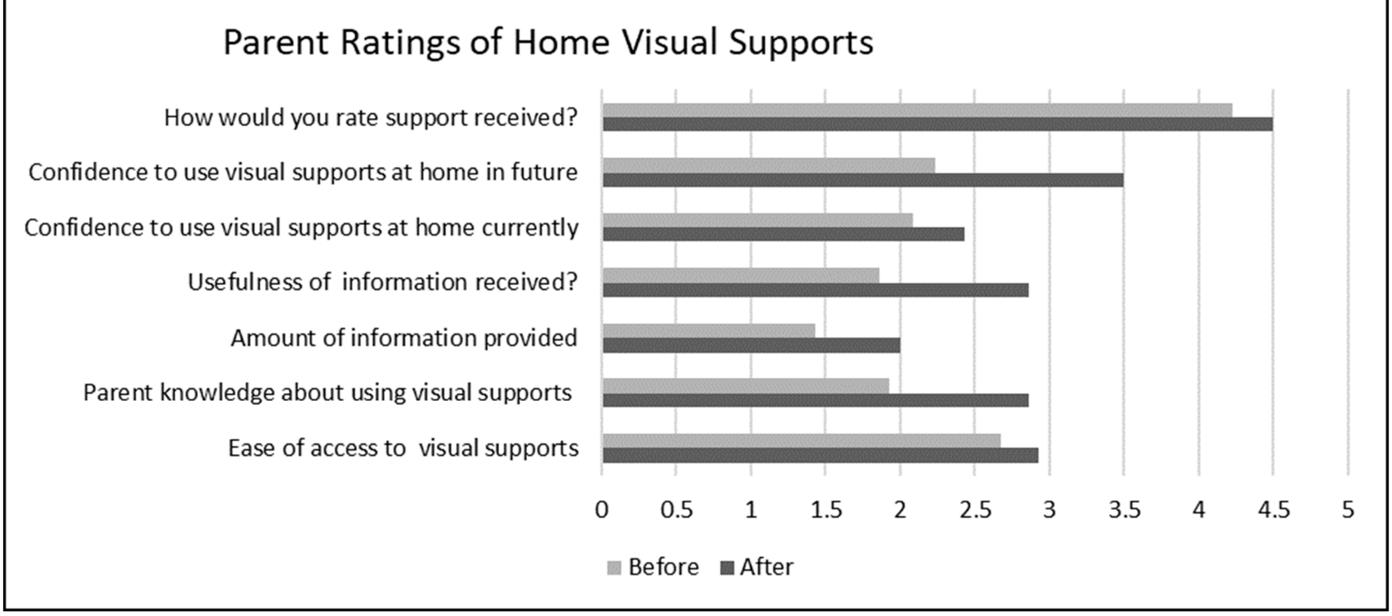

Rating key

*How would you **rate** this support received?*

Extremely Unhelpful	1
Unhelpful	2
Neither helpful nor unhelpful	3
Helpful	4
Extremely helpful	5

*How **confident** are you to use visual supports at home with your child at the moment?*

Not confident at all	1
A little confident	2
Quite confident	3
Very confident	4

*How **confident** are you to use visual supports at home with your child in the future?*

Not confident at all	1
A little confident	2
Quite confident	3
Very confident	4

*How **useful** has the information you have received?*

Not useful	1
Quite useful	2
Very useful	3

*Was the **amount** of information?*

Not enough	1
The right amount	2
Too much	3

*How much **knowledge** do you have about visual supports and how your child could use them?*

None	1
Some	2

A lot	3

*How **easy** is it to get the visual support symbols or resources that you would like?*

Very hard	1
Quite hard	2
Quite easy	3
Very easy	4
